# A National Pension Fund for Hospital Officials and Nurses

**Published:** 1887-03-19

**Authors:** 


### An Umbrella for a Rainy Day.

### A National Pension Fund for Hospital Officials and Nurses.

"He who does not teach his son a trade teaches him to be a thief," said an ancient Rabbi; and he who in youth makes no provision for old age, builds for himself a beggar's castle. When a dog has had his breakfast he probably has little care where his dinner is to come from, though even he will take pains to bury a bone for future use which may remain after the breakfast is finished. Man is a being who looks *before and after*—that is to say, the typical man is so. There are some people, both men and women, who look neither before nor after. "The prudent man looketh well to his going," said Solomon. He did not add anything about the prudent woman. He spoke, however, on another occasion of the *wise* woman: "Every *wise woman buildeth her house*: but the foolish plucketh it down with her hands." The least tedious of writers can hardly help moralising when he sits down to think of mankind in the mass. Christmas will come with its bills and its duns, but thousands of people make merry in November who have not put by a penny for either. Nothing is more certain than quarter-day, though there are householders by the score who provide neither rent nor rates.

**Provide or Perish.**

What has all this to do with hospitals? Not much with the buildings or committees or patients: but a great deal with the matrons, nurses, secretaries, and other officials. There is probably many a rainy day in store for most of them. Very few have private means, and not many are likely to have fortunes left. There is no prospect of wealth arising from their occupation. If a competency is to be acquired, it must, in most cases, be accumulated by the small and slow savings of a lifetime. Nothing is more sure than old age, except death. Hospital officials may restore others to health, but they can restore no one to youth, not even themselves. Of course there is no originality in these remarks; but the writer has no particular desire to be original or even amusing. His wish is to take his readers metaphorically by the button-hole and to "have it out with them" about a due provision for old age. It is quite evident, from the number and character of the letters we have received, that a good many hospital officials are looking forward to their declining years, and that the prospect is not particularly encouraging. At present little or no general provision has been attempted. But a body of twelve or fifteen thousand intelligent people ought, by putting their heads together, to be able to evolve a sound practical scheme whereby every self-denying nurse or other official may have full assurance of a modest competency when from age or debility she can work no more. For if nothing be done, what is the outlook for a good many? The workhouse! One shivers even to write it. Charity? It is all very well in the bulk; but when a little of it is required for a particular individual, it has a cold and icy feeling, which chills one to the very marrow. In this world self-help is the quality that demands and compels respect. Every beggar may kick a whining dog, but a dog with teeth and the pluck to use them is universally treated as a gentleman.

**A Retiring Pension Fund.**

A Retiring Pension Fund—that is the object to be aimed at. Where is it to come from? It may be benevolent, or it may be co-operative. For our part, we prefer the idea of co-operation and independence. If any generous outsider should initiate such a fund by the gift of a substantial sum, well and good; but that should not be waited for. Besides, there must be no promotion by favour. Every well-conducted nurse should be entitled at the proper time to demand her pension as a right. There is no crust so hard as the bread of charity, and no cake so sweet as that which is bought with honest earnings. Too much dependence upon charity is one of the curses of modern times. Let men and women learn to look to themselves for victory in the battle of life. That career must generally be regarded as a failure, which ends by living on means of other people's providing.

**Two Solutions Suggested.** There are two methods of securing the end to be aimed at. A new organisation may be created which shall prepare a scheme, receive monthly or quarterly payments, invest the moneys, and generally conduct the business of the fund. But what does this involve? It means central offices, with liberally paid officials, actuarial and other charges, and generally a large initial and annual expenditure. Mr. Burdett, at his own expense, had a scheme prepared by the late Mr. Walford, F.S.A., one of the most capable of British actuaries, and Mr. King, the able actuary of the Atlas Company, which only awaits the support of those interested to form the basis of a National Pension Fund for hospital officials and nurses, with a sound financial future. The contributions to the fund might be collected by the secretaries or lady superintendents of the various institutions, at the time the salaries are paid, and be remitted in one sum to the trustees, who would invest the money from time to time. Hospital committees might enable nurses to contribute their proportion by allowing in all cases a certain fixed sum for washing, the cost of which is now generally defrayed out of the nurse's own earnings. The Hospitals Association, by the appointment of Mr. Howard Collins, late assistant secretary to the Lock Hospital, as its secretary, is in a position to actively organise and carry through such a movement as this at the present time. If Mr. Newton H. Nixon and one other gentleman are prepared to act as Honorary Secretaries to a special committee for the purpose of organising such a scheme, and to ascertain the views and wishes of all those who are likely to be interested in it, the National Pension Fund might be an established success within a year from the present time. We shall be glad to publish any letters or suggestions which may be sent us in relation to these proposals; and meanwhile everybody who wishes to see the early establishment of such a fund should send in his or her name to Mr. Howard Collins, Secretary to the Hospitals Association, Norfolk House, Norfolk Street, Strand, London, W.C. By this means the cost of offices, actuaries, and other expenses of all kinds will be minimised, and security of the most undoubted character will be obtained.

**Its Vital Importance to all.** It is impossible to exaggerate the importance of such a scheme as this to hospital officials. The most sober and least imaginative of them cannot but see that some such means of livelihood must be provided for old age. The movement should be commenced at once. There is every reason for promptitude, and nothing to be gained by delay. The Hospitals Association would seem to be the most fitting body to take the initiative; and there need be no doubt that, should a strong feeling arise in its favour among hospital officials, a thoroughly practical and comprehensive scheme might speedily be carried to a successful issue.

